# The online mindset intervention ‘The Growth Factory’ for adolescents with intellectual disabilities: moderators and mediators

**DOI:** 10.1111/jir.12970

**Published:** 2022-09-01

**Authors:** F. Verberg, P. Helmond, R. Otten, G. Overbeek

**Affiliations:** ^1^ Pluryn Research & Development Nijmegen The Netherlands; ^2^ Research Institute of Child Development and Education University of Amsterdam Amsterdam The Netherlands; ^3^ Behavioural Science Institute Radboud University Nijmegen The Netherlands; ^4^ REACH Institute, Department of Psychology Arizona State University Tempe AZ USA

**Keywords:** Intellectual disabilities, Intervention, Mediation, Mental health, Mindset, Moderation, Perseverance

## Abstract

**Background:**

The online mindset intervention The Growth Factory (TGF) has shown promising effects—increasing growth mindsets and perseverance and decreasing mental health problems among youth with intellectual disabilities (ID). Studying moderators and mediators of intervention effects is essential to elucidate for whom and why TGF works. Using a randomised controlled trial design, we examined youth's baseline mindset, gender, age, level of ID and intervention satisfaction as moderators of TGF effects and examined whether the intervention effects of TGF on improvements in mental health were mediated by perseverance.

**Methods:**

The sample consisted of 119 participants with mild to borderline ID (*M*_*age*_ = 15.83; *SD* = 2.23), randomly assigned to the intervention (*n* = 60) or passive control group (*n* = 59). Participants reported mindsets, perseverance, internalising, externalising, attention and total mental health problems at pre‐test, at post‐test and at 3‐month follow‐up. Additionally, youth in the intervention group graded their satisfaction with a score at the end of each session.

**Results:**

Findings indicated that the effectiveness of TGF was not affected by participants' baseline mindsets, age and ID level. TGF was more effective in reducing internalising problems in girls and increasing perseverance in boys. In addition, in the intervention group TGF was more effective in improving internalising, externalising and total mental health problems for youth who reported higher levels of intervention satisfaction at post‐test. Finally, TGF indirectly decreased internalising and externalising problems at follow‐up through improvements in perseverance reported at post‐test.

**Conclusions:**

TGF offers a universal, ‘add‐on’ mindset intervention complementing usual care programmes. It improves mindsets, perseverance and mental health in youth with ID. Both practical and theoretical implications are discussed.

## Introduction

Youth with intellectual disabilities (ID) form an at‐risk population for a variety of outcomes. Specifically, compared with their peers without ID, youth with ID are more likely to experience emotional and behavioural problems (referred to as mental health problems), such as depression and aggression, and low self‐esteem (e.g. De Ruiter 2007; Einfeld *et al*., 2011). In addition, previous research demonstrated that youth with ID are more likely to endorse a fixed mindset—the belief that personal characteristics such as emotions, personality and intelligence are static and uncontrollable—compared with typically developing peers (Koestner *et al*. 1995; Baird *et al*. 2009; Verberg *et al*. 2019). However, a growth mindset—the belief that characteristics are malleable—has repeatedly been related to a variety of beneficial outcomes on mental health, prosocial behaviour and academic performance in youth with and without ID (e.g. Koestner *et al*. 1995; Baird *et al*. 2009; Yeager et al., 2013, 2016; Verberg *et al*. 2019; for a meta‐analysis, see Schleider *et al*., 2015).

Extensive evidence suggests that growth mindsets can be cultivated by mindset interventions (e.g. Schleider & Weisz 2016b, 2018; Yeager *et al*. 2016). These brief psychological interventions, generally one to eight sessions and executed face‐to‐face or using a computer program, convey messages about the malleability of personal attributes. The aim is to enhance growth mindsets and thereby positively impacting academic, social and psychological outcomes (Yeager & Walton 2011; Yeager & Dweck, 2012). Despite some recent unsupportive evidence and discussion about meaningful effect sizes (Sisk *et al*. 2018; Calvete *et al*. 2019; Foliano *et al*. 2019), promising effects of mindset interventions have generally been found (Yeager *et al*. 2014; Miu & Yeager 2015; Schleider & Weisz 2016b). Due to the increased risk of mental health problems, as well as more fixed‐oriented mindsets in youth with ID, a mindset intervention cultivating a growth mindset may be a successful way to reduce mental health problems in this at‐risk population.

Therefore, we developed the online mindset intervention The Growth Factory (TGF) for youth with ID. The six sessions of TGF are structured around the key growth mindset affirmations (e.g. Dweck 1999; Yeager & Walton 2011; Yeager *et al*. 2016) by emphasising (1) the potential for brain plasticity; (2) the assumption that one's characteristics (i.e. emotions, behaviours and skills) are malleable and have the potential to change; (3) that people are personally in charge of this process by teaching the formula for successful change: effort, changing strategies, help from others; and (4) that change is neither easy nor certain and may only happen over time—but is usually possible. Besides the use of animations, interactive assignments, movie clips of successful role models and ‘saying‐is‐believing’ exercises, TGF contains exercises based on the principles of cognitive behavioural therapy, role play, biweekly reminders and homework assignments (Aronson *et al*. 2002; Yeager & Walton 2011; Yeager *et al*. 2016).

In a previous study, we investigated the effectiveness of TGF in youths with ID (*N* = 119; 12–23 years) using a randomised controlled trial (RCT) (Verberg *et al*. 2021). Findings showed that TGF had positive effects among others on perseverance, internalising, attention and total mental health problems immediately after the intervention and on mindsets at 3 (intelligence) and 6 (intelligence, and emotion and behaviour) months follow‐up (Verberg *et al*. 2021). Besides obtaining insight into the effectiveness of TGF, it is important to create a more in‐depth understanding for *whom* TGF works and *how* TGF works. Therefore, the aim of the present study is to further elaborate on our previous results and examine whether (1) the effectiveness of TGF on mindsets, perseverance and mental health problems is moderated by youth's baseline mindsets, gender, age, ID level and intervention satisfaction and (2) the effect of TGF on mental health problems at 3‐month follow‐up (partially) runs via improvements in perseverance at post‐test. Logically, the current study used the same sample as the sample that was used in Verberg *et al*. (2021).

### Moderators of mindset intervention effects

Until now, most studies have mainly focused on the main effects of mindset interventions, but uncovering for whom mindset interventions are more beneficial has received little attention. When (certain) moderators were included within mindset studies among the general population, results demonstrated that individuals with low social dominance orientation (i.e. endorsing equality among social groups), poorly performing students and Black students with high expectations for future educational attainment benefitted the most from mindset interventions (Paunesku *et al*. 2015; Binning *et al*. 2018; Hoyt *et al*. 2018). Other studies found no moderating effects or mixed results of baseline mindset, age, gender and socioeconomic status (e.g. Aronson *et al*. 2002; Blackwell *et al*. 2007; Yeager *et al*. 2011, 2014; Miu & Yeager 2015; Paunesku *et al*. 2015; Sisk *et al*. 2018). To the best of our knowledge, previous studies have not examined moderators of the effectiveness of mindset interventions for youth with ID. In this study, we examined youth's baseline mindset (i.e. mindset of intelligence and mindset of emotion and behaviour), age, gender, level of ID and intervention satisfaction as moderators of the effectiveness of TGF.

First, it has been found that people who initially endorse a more fixed mindset, compared with those holding a more growth‐oriented mindset, generally benefit more from mindset interventions as it is hypothesised they have more to gain from learning the growth mindset affirmations (Blackwell *et al*. 2007; Yeager et al., 2014, 2016; Miu & Yeager 2015; Broda *et al*. 2018). In contrast, other studies demonstrated that mindset interventions help to reduce aggression, stress and health regardless of baseline mindset (Yeager *et al*. 2013; Broda *et al*. 2018). Therefore, in the present study, we will explore whether the effectiveness of TGF is moderated by baseline mindset.

Second, it has been suggested that intervention effects may be weaker for people with ID as thinking, processing information and learning occur at a slower rate (De Wit *et al*. 2011; Campbell *et al*. 2014). However, a meta‐analysis examining the moderating role of level of ID (i.e. mild (IQ 50–69), moderate (IQ 35–49), severe and profound (IQ <20) on intervention effects on challenging behaviour did not show an association with treatment effects (Heyvaert *et al*. 2010). It is important to note, however, that participants with borderline intellectual functioning (BIF) (IQ 70–85) were not included in this meta‐analysis (Heyvaert *et al*. 2010). Unlike other countries, in the Netherlands, individuals with borderline intelligence and with severe limitations in adaptive functioning are eligible for access to healthcare and special education systems for individuals with ID. Therefore, our study included youth with mild ID and BIF (IQ 50–85).

Third, although intervention satisfaction has not yet been examined as a moderator in studies concerning mindset intervention effects, positive associations between treatment satisfaction and treatment outcomes, such as fewer psychiatric symptoms and substance use problems, are commonly found (Zhang *et al*. 2008; Boden & Moos 2009). Therefore, we will investigate whether TGF intervention effects might be influenced by the satisfaction of participants with the intervention.

Finally, the literature currently is unclear whether gender and age actually moderate mindset intervention effects in the general population (e.g. Yeager *et al*. 2011, 2013; Paunesku *et al*. 2015). Despite mixed findings, it is relevant to include these variables in this first exploratively study into moderators of mindset intervention effects because of the heterogeneous population of people with ID.

### Mediators of mindset intervention effects

Although mindset interventions have shown to be effective in reducing mental health problems in youth with and without ID (Yeager *et al*. 2013, 2014; Schleider *et al*., 2015; Verberg *et al*. 2021), little is known about the mediating mechanisms that may more fully explain how these intervention effects come about. A previous study in the general population demonstrated that a mindset intervention decreased students' vulnerability to dysphoria through the reduction of self‐critical rumination (Baer *et al*. 2005). Another study among participants without ID showed that a mindset intervention strengthened youths' capacity to recover from stress through increases in a growth‐oriented mindset and perceived control (Schleider & Weisz 2016b). One possible mediator that has not yet been examined is perseverance. We decided to focus on the mediator perseverance because of the significant intervention effects of TGF on perseverance at post‐test.

Perseverance refers to an array of self‐regulatory processes in terms of attributions and reactions to effort, failure and challenges. As previously stated in the literature, mindset and perseverance are closely related concepts (Burnette *et al*., 2013; Mrazek *et al*. 2018; Sisk *et al*. 2018). In particular, when people believe in the malleability of their traits, they will be more eager to learn and practice, embrace challenges as learning opportunities and exert effort in the face of setbacks as they attribute failure as a result of insufficient effort or strategy. People with a fixed mindset, on the other hand, will interpret failure as a lack of ability and will generally feel helpless to change their circumstances (e.g. Blackwell *et al*. 2007; Duckworth *et al*. 2007; Burnette *et al*., 2013; Mrazek *et al*. 2018).

Mindset interventions have positively affected perseverance in the general population (Dweck & Leggett, 1988; Blackwell *et al*. 2007; Yeager *et al*. 2016; Burgoyne *et al*., 2018; Mrazek *et al*. 2018). Recently, our prior work extend these findings by showing that TGF was also effective in improving perseverance among youth with ID (Verberg *et al*. 2021). The effect on perseverance might be explained by TGF's explicit focus on the potential benefits of effort in learning and changing emotions and behaviours, finding an effective strategy and persisting despite setbacks. Therefore, perseverance might play a key role as mechanism in TGF's effects on mental health problems. In the present study, we will therefore examine whether the reductions in mental health problems are obtained through improvements in perseverance.

### Present study

The first objective of the present study was to examine the moderating role of baseline mindsets, gender, age, level of ID and intervention satisfaction on immediate effects of the online mindset intervention TGF on mindsets (i.e. mindset of intelligence and mindset of emotion and behaviour), perseverance and mental health (i.e. internalising, externalising, attention and total mental health problems). We hypothesised that adolescents with a more fixed mindset at baseline, with BIF and with higher intervention satisfaction scores would show larger increases in growth mindsets and perseverance and larger decreases in mental health problems compared with adolescents with a more growth mindset at baseline, with mild ID and with less satisfaction with the intervention. In addition, we explored whether gender and age moderated the intervention effects. The second objective of the current study was to examine perseverance as a mediator of TGF effects on mental health outcomes in ID youth. For this analysis, we also used the follow‐up assessment.

## Method

### Design

We conducted an RCT. Findings on the direct effects of TGF are published elsewhere (Verberg *et al*. 2021). Prior to data collection for the initial RCT (Verberg *et al*. 2021), a power analysis (two‐tailed, alpha 0.05, statistic power 0.80) based on a three‐measurement design indicated that 106 participants were necessary to obtain significant results for the RCT. In addition, prior to main analyses, we checked possible baseline differences in demographic variables and study outcomes between the intervention and control group using independent‐sample *t*‐tests and chi‐square tests. Ethical approval was granted by the Ethics Review Board of the University of Amsterdam (2015‐CDE‐4518), and the study was registered in the Dutch Trial Register for RCTs (www.trialregister.nl; NTR5460*)*. A comprehensive description and tables and figures of the trial design, participants, procedure and measures can be found in previous publications (Verberg *et al*. 2018, 2019, 2021).

The analyses presented in *this* paper focus on the effects at post‐test (moderation) and 3‐month follow‐up (mediation).

### Participants

**Figure 1:**
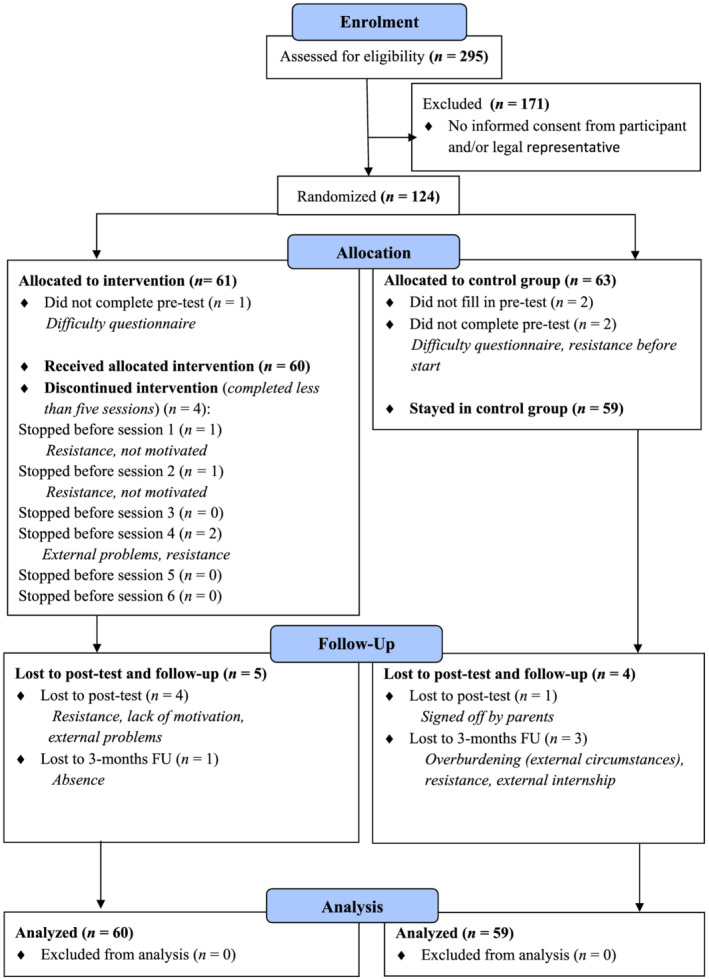
Flow diagram. [Colour figure can be viewed at wileyonlinelibrary.com]

Participants were recruited from a residential care organisation and six special education schools for youth with ID and co‐morbid physical and/or psychiatric problems in the Netherlands. Data collection took place in five rounds between October 2015 and 2017 (pre‐Covid). In total, 124 youths were included in the present study, but five participants dropped out before the pre‐test. One participant showed resistance before start, and four participants were unable to fill in the questionnaires due to their ID. The final sample consisted of 119 participants with a mean age of 15.83 years (*SD* = 2.23) and an average intelligence score of 66.41 (range 50–85). In addition to an ID, the majority of the participants (92.4%) were diagnosed with co‐morbid problems including a physical disability (e.g. cerebral palsy and spina bifida), psychiatric disorder (e.g. attention deficit hyperactivity disorder) or both (see Table 1). Attrition was low with 9 (7.6%) participants dropping out of the study until 3‐month follow‐up. Participant flow is shown in Fig. 1.

**Table 1:** Participants' characteristics and group differences at pre‐test

Variable	Intervention *(n* = 60)		Control *(n* = 59)		Total (*N* = 119)		Statistics
	*n*	*%*	*n*	*%*	*N*	*%*	*t*
Gender							*t*(117) = −.08, *P = 0.94*
Male	35	58.3	34	57.6	69	58	
Female	25	41.7	25	42.4	50	42	
TIQ − M (SD)	66.9 (10.03)		65.9 (9.08)		66.4 (9.54)		*t*(117) = −0.56, *P = 0.58*
Age − M (SD)	15.9 (2.25)		15.8 (2.22)		15.8 (2.23)		*t*(117) = −0.38, *P =* 0*.70*
Age groups							*t*(117) = −0.34, *P = 0.74*
Early ad (<15 yrs)	22	36.7	24	40.7	46	38.7	
Mid‐late ad (>15 yrs)	38	63.3	35	59.3	73	61.3	
Level of ID							*t*(117) = −0.34, *P = 0.74*
Mild ID	41	68.3	42	71.2	83	69.7	
Borderline IF	19	31.7	17	28.8	36	30.3	
Co‐morbidity	53	88.3	57	96.6	110	92.4	
Physical disability	36	67.9	41	71.9	77	70.0	*t*(117) = .43, *P = 0.67*
Psychiatric problem	5	9.4	8	14.0	13	11.8	*t*(117) = −0.15, *P = 0.88*
Multiple	12	22.6	8	14.0	20	18.2	*t*(117) = −0.94, *P = 0.35*

Abbreviations: ad, adolescence; yrs, years; ID, intellectual disability; IF, intellectual functioning.

Multiple = physical disability and psychiatric problem.

### Procedure

Participants were recruited by treatment coordinators of the institute and the school psychologists. Inclusion criteria were youth between 12 and 23 years old having an IQ score within the mild (IQ 50–69) or borderline (IQ 70–85) ID range. Exclusion criteria were severe emotional problems, such as extreme aggression problems or an acute unstable mental condition, hindering participation in the study. Informed consent was obtained from all participants and their legal representatives. After randomisation, participants were informed about the allocation to the intervention or control group by their mentor. Self‐report questionnaires and additional information from case files (i.e. age, gender, diagnoses and IQ) were used. Questionnaire assessments were conducted at pre‐test, post‐test and a follow‐up at 3 months after the intervention. Questionnaires were completed on a computer, and all youth were guided individually by a trained research assistant. Research assistants read all questions aloud, checked participants understanding, used standardised clarification and provided help if needed. For 6 consecutive weeks, youths in the intervention group participated individually in six sessions lasting 25–45 min as an addition to their usual care programme. Participants assigned to the control group attended the school curriculum or care as usual.

### Measures

Based on the Dutch guideline for developing, adjusting and conducting diagnostic instruments for people with ID (Douma *et al*., 2012), the following questionnaire adjustments were made to reduce the complexity of the item content and task load for participants: (1) difficult words and sentences were simplified or rephrased, (2) answering categories were unified into one format ranging from ‘completely untrue’ to ‘completely true’, and (3) answering categories were supported with coloured emoticons.

#### Mindset and perseverance

Beliefs about the malleability of emotion and behaviour and intelligence and participants' perseverance (i.e. attributions and responses to effort, failure and challenges) were assessed using the Mindset and Perseverance Questionnaire (MPQ; Verberg *et al*. 2019). The MPQ consists of two parts measuring mindset of emotion and behaviour and mindset of intelligence (nine items, e.g. ‘I can learn to control how I feel’ and ‘How smart I am is sort of fixed’) and adolescents' perseverance (nine items, e.g. ‘Practising a lot is useless’). Items were scored on a 5‐point Likert scale, and all fixed mindset statements were reverse‐scored such that higher scores indicated a growth mindset and more perseverance. Reliability coefficients of the MPQ among youth with ID and youth with special needs have previously been reported to range from modest to adequate (Verberg *et al*. 2019, 2021; Helmond *et al*. 2022). In the present study, Cronbach's alphas for pre‐test, post‐test and follow‐up at 3 months respectively showed α = 0.61, 0.56 and 0.60 for mindset of emotion and behaviour, α = 0.68, 0.71 and 0.72 for mindset of intelligence and α = 0.74, 0.73 and 0.82 for perseverance.

#### Mental health problems

Mental health problems were assessed using the Dutch version of the Brief Problem Monitor‐Youth (BPM‐Y; Achenbach *et al*. 2011; Verhulst & Van der Ende 2013). The BPM‐Y contains 19 items measuring internalising problems, externalising and attention problems (e.g. ‘I feel unhappy, sad or depressed’, and ‘I threat other people’). The sum of the items yields a total problem score. Previous research showed sufficient to good reliability of this instrument among youth with ID and youth with special needs (Verberg *et al*. 2019, 2021; Helmond *et al*. 2022). In the present study, Cronbach's alphas for the three measurement points showed α = 0.76, 0.78 and 0.74 for internalising problems, α = 0.77, 0.77 and 0.78 for attention problems, α = 0.73, 0.73 and 0.74 for externalising problems and α = 0.85, 0.87 and 0.85 for the total problems scale.

#### Intervention satisfaction

At the end of each session, participants in the intervention group were asked to grade their satisfaction with a score from *1* (*very low*) to *10* (*very high*). The mean of the satisfaction grade scores of the six sessions was taken to construct an overall mean intervention satisfaction grade.

### TGF intervention

TGF builds on scientific research on mindset theories and mindset interventions by Carol Dweck and David Yeager (Dweck 1999; Yeager *et al*. 2013, 2016). The received mindset materials from Yeager and colleagues (Yeager *et al*. 2013, 2016; Paunesku *et al*. 2015) were adapted with youths with ID and professionals using the guideline for effective interventions for people with intellectual disabilities (De Wit *et al*. 2011). A professional graphic designer animated the delivery of content of the intervention. By the online approach, we were able to use visual and auditory support and provide a structured learning environment with the possibilities to repeat parts of a session or make use of extra advice when desired. For a detailed description of the development, structure and content of the intervention, see our previous studies (Verberg *et al*. 2018, 2019, 2021).

### Statistical analyses

To investigate the moderating effects of baseline mindset (i.e. mindset of emotion and behaviour and mindset of intelligence), gender, age, level of ID and intervention satisfaction (grade) on all outcome variables at post‐test, we conducted separate multivariate linear regression analyses controlling for gender, total IQ and age, with a two‐step approach. In each separate analysis, in the first step, condition and the moderation variable of interest were included in the regression model together with covariates. In a second step, the centred cross‐product of condition and the moderators of interest were added to the model to test moderation (e.g. condition * baseline mindset of intelligence). When significant, the interaction was plotted further to gain insight into the precise direction of the moderating effect. With respect to potential moderation effects of satisfaction, moderation only focused on those youth who participated in TGF (e.g. internalising problems * satisfaction). We created a low and high satisfaction group by using a median split. To test whether improvements in mental health outcomes at 3‐month follow‐up were mediated by changes in perseverance at post‐test, we conducted multivariate linear regression analyses using Mplus Version 7 (Muthén & Muthén 1998‐2012) while controlling for gender, age, total IQ and baseline perseverance. Mediation was tested using indirect effects (by using the MODEL INDIRECT command) and bootstrapping with 5000 random draws (MacKinnon *et al*. 2007).

The ‘CLUSTER’ command was used to take in account the non‐independence of data due to youths receiving school or care at different treatment locations, which could otherwise artificially inflate the standard errors of the parameter estimates. As a consequence, we used the MLR estimator. MLR (Maximum likelihood with robust standard errors) is a maximum likelihood method that takes clustering of cases into account and estimates parameters with standard errors and a chi‐square test statistic (when applicable) that are robust to non‐normality and non‐independence of observations when used with ‘TYPE = COMPLEX’. This maximum likelihood method, by which parameters are estimated using available data with robust standard errors, is also used in strategies to handle missing data. Specifically, after the parameters are estimated using the available data, missing data are estimated based on the parameters that have just been estimated (Muthén & Muthén 1998‐2012). The Benjamini–Hochberg false discovery rate correction was used to correct for chance capitalisation across all tests (Benjamini & Hochberg 2005). The *P* values after this correction are presented in the ‘Results’ section. All analyses were based on an intention‐to‐treat sample (i.e. including data from all participants and whether or not they completed all sessions and assessments). In addition, a completers‐only analysis was conducted (i.e. involving only adolescents in the intervention group who completed five or six sessions; *n* = 56). The analyses were reported in accordance with the CONSORT statement (Schulz *et al*. 2010). See the CONSORT‐SPI 2018 checklist included in Data S1.

## Results

### Preliminary results

At baseline, participants in the intervention and control group did not significantly differ on demographic (see Table 1) and outcome variables (all *P* > 0.10; Verberg *et al*. 2021). In addition, no outliers were found and analyses with completers‐only revealed similar results. Table 2 shows descriptive statistics for the outcome variables mindsets, perseverance, and mental health problems at pre‐test and post‐test. For convenience of the reader, Table 3 shows the direct effects of TGF on mindsets, perseverance and mental health problems at post‐test (Verberg *et al*. 2021).

**Table 2:** Means and standard deviations for intervention and control group at pre‐test and post‐test

	Intervention group^a^				Control group^b^			
Variable	*T0*		*T1*		*T0*		*T1*	
	*M*	*SD*	*M*	*SD*	*M*	*SD*	*M*	*SD*
*Mindset*								
Mindset EB	3.75	0.61	3.83	0.45	3.59	0.61	3.78	0.63
Mindset IQ	3.11	0.76	3.30	0.80	3.02	0.96	3.05	0.86
*Perseverance*	3.76	0.57	4.06	0.41	3.86	0.54	3.84	0.55
*Mental health problems*								
Total	1.66	0.36	1.52	0.35	1.59	0.32	1.58	0.37
Internalising	1.66	0.47	1.47	0.40	1.58	0.46	1.57	0.50
Externalising	1.50	0.43	1.42	0.38	1.44	0.35	1.41	0.40
Attention	1.86	0.49	1.68	0.46	1.77	0.50	1.79	0.50

EB, emotion and behaviour; IQ, intelligence.

^a^ T0 *n* = 60; T1 *n* = 55; T2 *n* = 55.

^b^ T0 *n* = 59; T1 *n* = 58; T2 *n* = 55.

**Table 3:** Effects of The Growth Factory at post‐test

	Pre‐test–post‐test		
Variable	*B*	*SE B*	*P*
Mindset			
Mindset EB	−0.027	0.079	0.730
Mindset IQ	0.104	0.065	0.108
Perseverance	0.225	0.077	0.000
Mental health problems			
Total	−0.164	0.032	0.000
Internalising	−0.161	0.045	0.000
Externalising	−0.043	0.038	0.263
Attention	−0.167	0.048	0.000

B, standardised regression coefficient; EB, emotion and behaviour; IQ, intelligence.

*P* values are corrected with Benjamini–Hochberg false discovery rate correction.

### Moderators of intervention effects

The main analyses of this study pertained to the analysis of the potential moderators and mediators on the effectiveness of TGF. Table 4 shows all findings for the interaction terms on mindsets, perseverance and mental health problems. Because of the large number of interactions that were tested, we here *only* report the findings for the significant interactions. 1

**Table 4:** Moderating variables of intervention effects on mindsets, perseverance and mental health problems

		Pre‐test–post‐test		
Outcome	Interaction	*B*	*SE*	*P*
Mindset EB	Condition × total IQ	−0.044	0.126	0.725
	Condition × mindset EB T0	−0.073	0.123	0.556
	Condition × mindset IQ T0	−0.038	0.080	0.637
	Condition × age	0.090	0.071	0.207
	Condition × gender	0.031	0.095	0.745
Mindset IQ	Condition × total IQ	0.122	0.122	0.237
	Condition × mindset EB T0	0.109	0.078	0.161
	Condition × mindset IQ T0	0.017	0.017	0.715
	Condition × age	0.022	0.049	0.655
	Condition × gender	−0.042	0.068	0.536
Perseverance	Condition × total IQ	0.065	0.075	0.386
	Condition × mindset EB T0	−0.023	0.072	0.751
	Condition × mindset IQ T0	−0.018	0.070	0.795
	Condition × age	0.013	0.080	0.872
	Condition × gender	0.084	0.024	0.000
Total mh problems	Condition × total IQ	−0.014	0.039	0.721
	Condition × mindset EB T0	0.006	0.040	0.989
	Condition × mindset IQ T0	−0.001	0.073	0.890
	Condition × age	−0.003	0.039	0.942
	Condition × gender	0.022	0.026	0.401
Internalising problems	Condition × total IQ	−0.023	0.031	0.452
	Condition × mindset EB T0	−0.023	0.061	0.709
	Condition × mindset IQ T0	0.046	0.057	0.422
	Condition × age	−0.038	0.061	0.532
	Condition × gender	0.080	0.026	0.002
Externalising problems	Condition × total IQ	−0.001	0.103	0.237
	Condition × mindset EB T0	0.025	0.066	0.703
	Condition × mindset IQ T0	0.090	0.054	0.098
	Condition × age	−0.004	0.038	0.910
	Condition × gender	0.008	0.039	0.835
Attention problems	Condition × total IQ	0.003	0.033	0.973
	Condition × mindset EB T0	−0.037	0.084	0.659
	Condition × mindset IQ T0	−0.106	0.106	0.317
	Condition × age	0.014	0.051	0.781
	Condition × gender	−0.006	0.049	0.904
Satisfaction (intervention group)	Mindset EB × satisfaction	0.273	0.148	0.065
	Mindset IQ × satisfaction	0.289	0.171	0.090
	Perseverance x satisfaction	0.169	0.186	0.364
	Total mh problems × satisfaction	−0.244	0.072	0.001
	Internalising problems × satisfaction	−0.333	0.088	0.000
	Externalising problems × satisfaction	−0.156	0.047	0.001
	Attention problems × satisfaction	0.005	0.025	0.825

*B*, standardised regression coefficient; mh, mental health.

Please note that each line refers to a separate regression analysis. *B* = standardised regression coefficient. *P* values are corrected with Benjamini–Hochberg false discovery rate correction.

**Figure 2:**
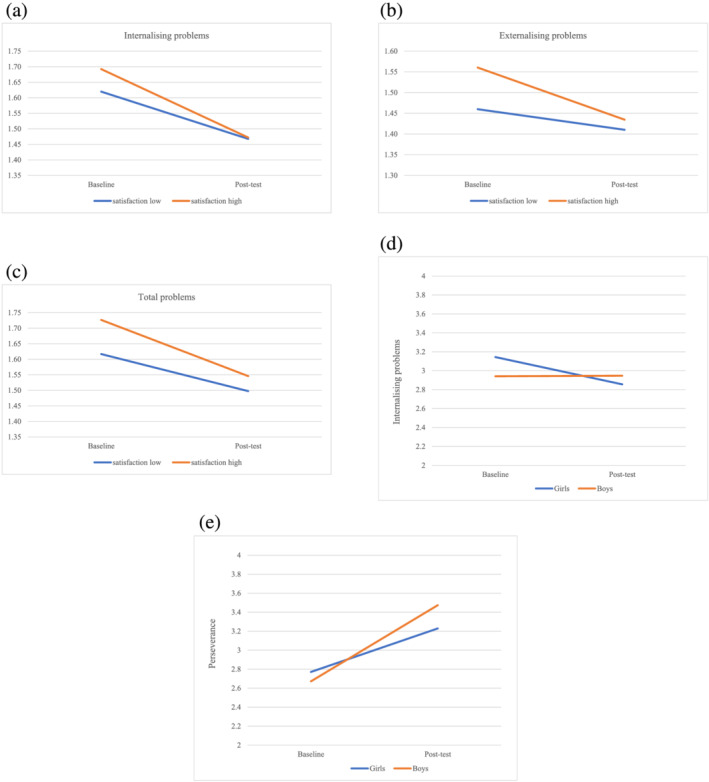
(a) Intervention satisfaction as a moderator of intervention effects on internalising problems. (b) Intervention satisfaction as a moderator of intervention effects on externalising problems. (c) Intervention satisfaction as moderator of intervention effects on total mental health problems. (d) Gender as moderator of intervention effects on internalising problems. (e) Gender as moderator of intervention effects on perseverance. [Colour figure can be viewed at wileyonlinelibrary.com]

As is shown, baseline mindsets, age and level of ID did not moderate intervention effects on mindsets, perseverance and mental health outcomes in the intervention and control group at post‐test. Intervention satisfaction and gender *did* have an effect on the effectiveness of the intervention. Specifically, as shown in Fig. 2a–c, intervention effects with respect to reducing internalising, externalising and total mental health problems were stronger for adolescents who were more satisfied about the intervention compared with those with lower satisfaction scores. In addition, TGF was more effective in reducing internalising problems in girls, and increasing perseverance in boys (Figures 2d,e).

### Mediation effects

Finally, we examined whether perseverance could account for the intervention effect of TGF on mental health problems. Our previous study (Verberg *et al*. 2021) showed that TGF had a direct effect on internalising problems. Moreover, the present study demonstrated that the effect of TGF was partially mediated by perseverance (*B*_*internalising*_ = −0.036, 95% CI [−0.071, −0.001], *SE* = 0.018, *P* = 0.046). TGF affected internalising problems at 3‐month follow‐up via perseverance at post‐test.

**Figure 3:**
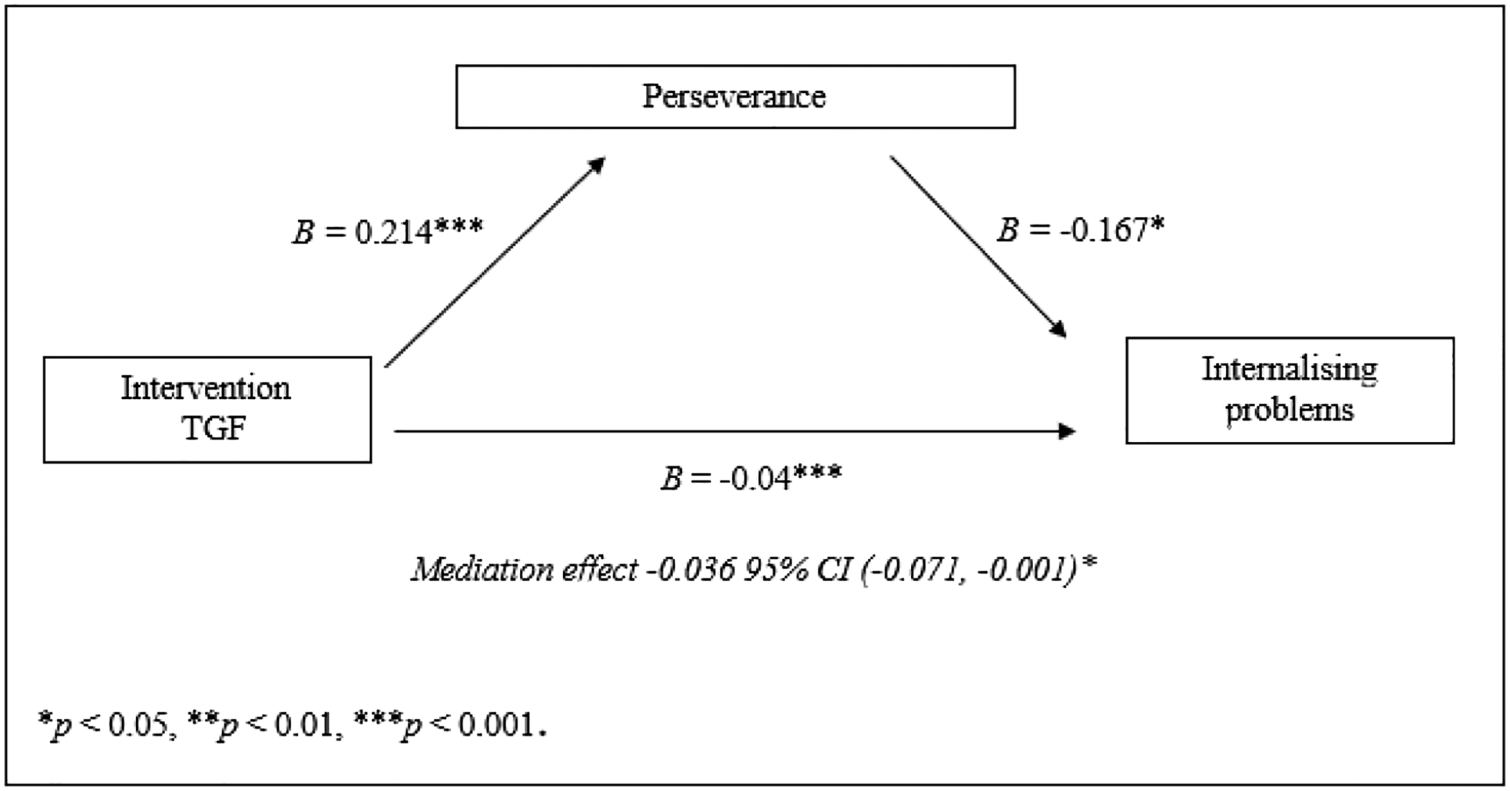
Mediation effect of TGF on internalising problems at follow‐up via perseverance at post‐test.

**Figure 4:**
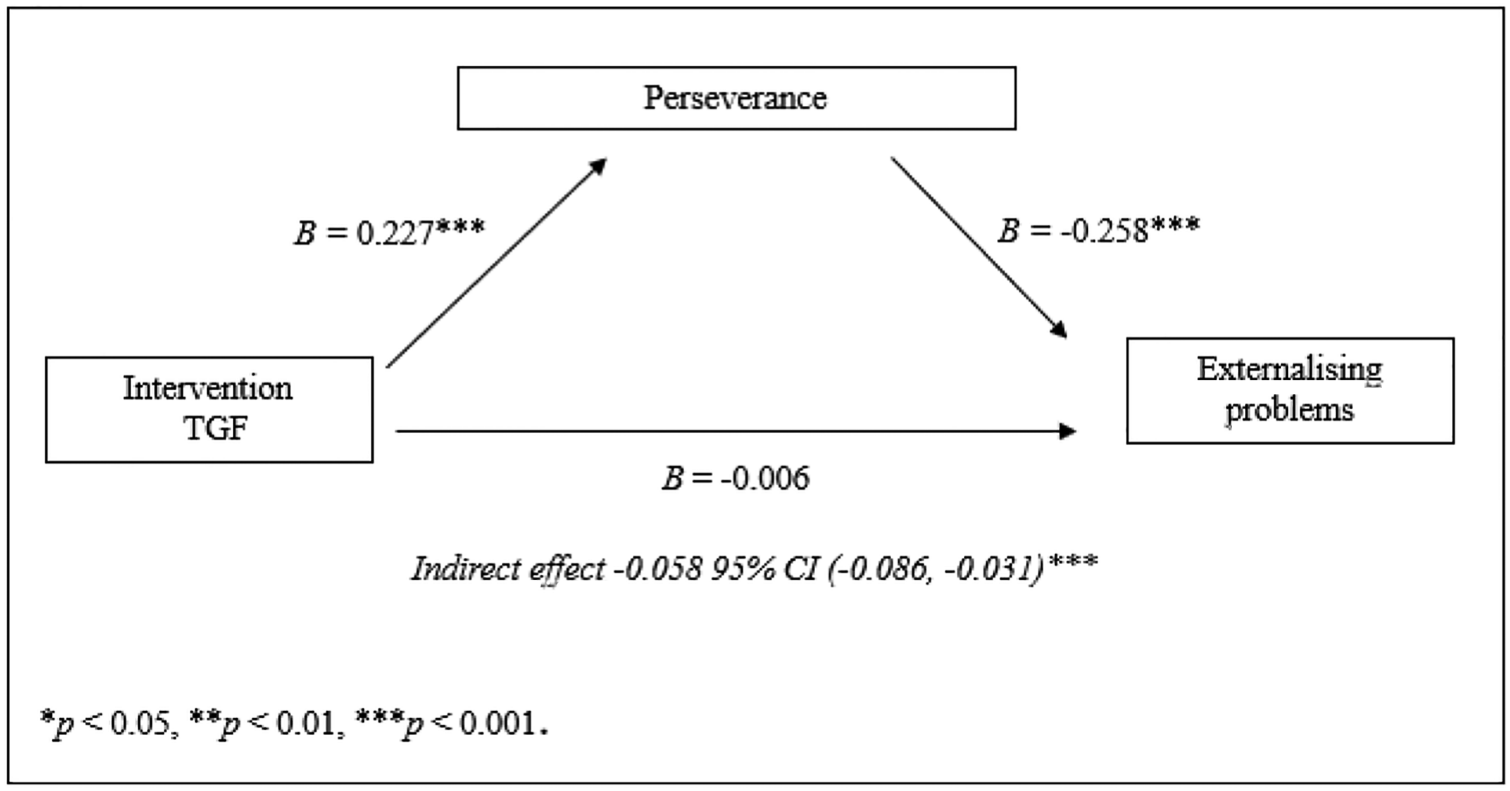
Indirect effect of TGF on externalising problems at follow‐up via perseverance at post‐test.

Furthermore, our previous study (Verberg *et al*. 2021) demonstrated that TGF did not have a direct effect on externalising problems; however, our findings showed support for an indirect effect in which TGF affected externalising problems at 3‐month follow‐up via perseverance at post‐test [*B*_*externalising*_ = −0.058, 95% CI (−0.086, −0.031), *SE* = 0.014, *P* < 0.001]. Thus, TGF was associated with improved perseverance, in turn decreasing internalising and externalising problems (see Figures 3,4).

## Discussion

Previous research showed that TGF, an online mindset intervention developed for youth with ID, improves growth mindsets and perseverance and decreases mental health problems in this at‐risk population (Verberg *et al*. 2021). However, it was not yet clear for *whom* and *how* TGF works. Therefore, the objective of the present study was to investigate baseline mindsets, gender, age, level of ID and intervention satisfaction as moderators of the TGF effectiveness and to examine whether the intervention effects of TGF on improvements in mental health were mediated by perseverance. Results indicated that TGF was similarly effective for adolescents regardless of age, with less or more fixed mindsets at baseline, and for adolescents with different levels of intellectual functioning. However, we did find that participants in the intervention group who were more satisfied with the intervention showed larger reductions of internalising, externalising and total mental health problems compared with participants who were less satisfied with the intervention. In addition, girls benefitted more from TGF compared with boys in reducing internalising problems. In contrast, TGF was more effective in increasing perseverance in boys. Moreover, we found that TGF indirectly reduced internalising and externalising problems at 3‐month follow‐up through improvements in perseverance.

Our findings are in line with existing evidence that mindset interventions lead to improved mental health across different subgroups (e.g. Yeager *et al*. 2011; Miu & Yeager 2015; Paunesku *et al*. 2015; Schleider *et al*. 2020). A possible explanation for this might be that TGF maximises the fit between participants with diverse characteristics and the intervention. Purposefully, in developing TGF, special care was taken to increase the likelihood that participants would identify with one of the avatars or ‘buddies’ by creating avatars with different characteristics and by creating role models in the video clips whose stories and struggles matched those oftentimes reported by youth with ID and professionals (Yeager & Walton 2011; Binning *et al*. 2018). Additionally, youth were allowed to personalise their responses (e.g. by choosing their own topic in an assignment), so intervention materials evoked the intended experience in the way that was most relevant to them (Yeager & Walton 2011). Finally, the online approach provided the opportunity to adjust the level of support and repetition increasing the probability to address participants' individual information processing needs (De Wit *et al*. 2011).

This study confirms the relevance of considering intervention satisfaction as a moderator of intervention effects, as youth in the intervention group who were more satisfied with TGF demonstrated larger effects in reducing internalising, externalising and total mental health problems. This finding is in line with previous research, suggesting that intervention satisfaction might be a good proxy for engagement in and positive reactions to an intervention contributing to treatment outcomes (Dearing *et al*. 2005; Boden & Moos 2009). In addition, finding ways to improve intervention satisfaction might contribute to the effectiveness of interventions. Notably, with respect to gender, TGF was more effective in increasing perseverance in boys and in reducing internalising problems in girls. This could be explained by the contrasting coping strategies boys and girls have with regard to mental health problems (Kelly *et al*., 2008; Schleider & Weisz 2016a). For example, girls tend to engage more in self‐critique and rumination, whereas boys are more eager to blame others to reduce negative feelings (Schleider & Weisz 2016a). Therefore, for boys, the TGF key messages that encourage effort and that teach that they are personally in charge of changing their emotions and behaviours may have resulted in increased perseverance, whereas for girls learning about the malleability of personal traits may have resulted in opportunities to change internal states and self‐critique.

Interestingly, although there was no direct effect of TGF on externalising problems, TGF did indirectly reduce externalising problems through improvements in perseverance. One potential explanation is that teaching people to tolerate challenges and failures towards goal achievement increases their awareness of how their daily actions and habits are instrumental in achieving goals and changing emotions and behaviour (Zainal & Newman, 2019). Moreover, TGF encourages participants to cope with obstacles and challenges by putting in effort and exploring different strategies and to ask for help. This may have helped TGF participants to disconfirm negative expectancies and to better cope with distress (Zainal & Newman, 2019).

### Strengths and limitations

The present study has several limitations. First, our findings should be considered with some caution, as the relatively small sample size may have resulted in a lack of sufficient power and sensitivity to detect potential interaction effects. Especially, the analysis of the moderating variable intervention satisfaction was conducted only for the experimental group (*n* = 60). In addition, at pre‐test, five participants with mild ID dropped out due to difficulties with the questionnaires, potentially contributing to a selection bias concerning the moderating variable of level of ID. Furthermore, research assistants were not blinded for condition. This may have caused a potential source for bias at the different measurement points for participants in the intervention group. Moreover, we cannot fully eliminate the risk that the responses of participants in the intervention group on the intervention satisfaction measure were partially due to their desire to please the trained researchers. In addition, the subscale mindset of emotion and behaviour suffered from modest internal reliability, and therefore, caution is needed when interpreting the results. Another limitation is that we, in our attempt to create a more in‐depth understanding why TGF works, only examined one mediator of TGF on mental health problems. Finally, we cannot rule out that effect sizes would have been different if we had used an active control group. However, previous research showed that a mindset intervention was more effective than both a passive (no intervention) and an active control condition (Aronson *et al*. 2002; Yeager *et al*. 2013). Despite these limitations, the current study has several strengths. In particular, this research is a unique first attempt to expand the understanding of the working mechanisms of a mindset intervention for youth with ID. Specifically, examining perseverance as a potential mediating mechanism underlying mindset intervention effects had not been done until now. Also, this study was the first to systematically examine who benefits the most from TGF by testing several moderators. A particular strength is the stringent research design we used, a full‐scale randomised trial with repeated measurements with a sample of youth with ID from a ‘real‐world’ setting, including special education and residential care. Finally, due to the online approach, dissemination and implementation of TGF will be efficient and cost‐effective, and therefore, TGF will be able to be used on large scale.

### Clinical implications

The outcomes of the moderator analyses demonstrate that the online mindset intervention TGF is similarly effective for adolescents with diverse characteristics, with two exceptions regarding gender. Therefore, TGF can be used for a broad range of youth with ID and delivered widely across special education schools and residential care. In addition, the results underline the importance of both systematically monitoring and boosting intervention satisfaction in TGF and, might we speculate, in interventions in general. Clearly, there is a great need for more specific information about which intervention content, processes and types of interactions with the trainer influence satisfaction, suggesting the use of feedback‐informed treatment (Miller *et al*., 2016). Additionally, feedback informed treatment as well as new intervention content designed to maximise relevance for youth experiencing externalising and internalising problems (e.g. even more specific social narratives of role models) may contribute to higher satisfaction levels among youths participating in the intervention, subsequently enhancing intervention effects (Binning *et al*. 2018; Schleider & Weisz, 2018; Limeri *et al*. 2020). Moreover, an additional program specifically developed for family members, mentors and clinicians might play a role in generalising the effects in the long run (Dweck & Yeager 2019; Yeager & Dweck 2020). Finally, TGF indirectly reduced internalising and externalising problems through improvements in perseverance, and this suggests that practitioners may be especially successful in decreasing those problems in youth with ID, by primarily encouraging effort, and offering strategies that help to persevere in the face of obstacles and challenges.

### Future research

Further research is needed that is designed and powered to undertake moderator analyses to replicate our findings in order to allow for more firm conclusions and deepen the understanding of the moderating and mediating mechanisms of TGF and mindset interventions in general. Because our knowledge of these underlying mechanisms is still very limited, we encourage future research to continue to explore different mechanisms that might explain the effects of TGF. For example, the therapeutic relationship may partially account for the effect of TGF on internalising problems, because alliance has shown to play a key role in enhancing treatment outcomes (e.g. Shirk & Saiz, 1992). Moreover, RCTs with an active control group may help to identify more specific elements of TGF that induce effects (Schleider & Weisz, 2018). Better insight into the active intervention ingredients may also help to customise TGF to improve its effectiveness. Moreover, future research should include additional interim assessments during the course of the intervention for a more detailed insight into the process of change. Furthermore, it seems relevant to look into the bidirectional associations between perseverance and mental health problems, as multiple theories have proposed how deficits in behavioural strategies, such as persevering despite setbacks, can precede, be a consequence of, or relate dynamically to mental health problems (Zainal & Newman, 2019). Finally, further investigations into other moderators, such as initial levels of perseverance and mental health problems, and parental support and coping style, might yield important discoveries (Zhou *et al*. 2007; Zainal & Newman, 2019).

## Conclusion

This study demonstrated that the online mindset intervention TGF appears to be effective for a variety of youth with ID in increasing mindsets and perseverance and reducing mental health problems. Furthermore, TGF was successful in decreasing externalising and internalising problems by promoting perseverance among youth with ID. Overall, our findings suggest that TGF can be used as a universal, ‘add‐on’ mindset intervention, complementing usual care programs improving growth mindsets, perseverance and mental health in ID youth.

## Source of funding

This study was supported by the Mr. F. Couvee‐Stichting, Johanna Kinderfonds and Stichting Rotterdams Kinderrevalidatie Fonds Adriaanstichting, University of Amsterdam, and Pluryn. None of these contributors had any role or authority in study design (e.g. data collection, management, analysis and interpretation of data) or writing of the manuscript.

## Conflict of interest

The authors FV and PH were developers of the online intervention TGF.

## Ethics statement

The Ethics Review Board of the University of Amsterdam in The Netherlands has approved this study (2015‐CDE‐4518). Written Informed consent was obtained from all individuals included in the study. Moreover, the study is registered in the Dutch Trial Register for RCTs (NTR5460).

## Supporting information


**Data S1.** Supporting Information

## Data Availability

Data are available on request.

## Footnotes

^1^
A complete overview of each separate regression analysis, and its findings can be obtained from the first author.

## References

[jir12970-bib-0001] Achenbach T. M. , McConaughy S. H. , Ivanova M. Y. & Rescorla L. A. (2011). Manual for the ASEBA Brief Problem Monitor (BPM). Burlington, VT: University of Vermont, Research Center for Children, Youth, and Families.

[jir12970-bib-0002] Aronson J. , Fried C. B. & Good C. (2002) Reducing the effects of stereotype threat on African American college students by shaping theories of intelligence. Journal of Experimental Social Psychology 38, 113–125.

[jir12970-bib-0003] Baer A. R. , Grant H. & Dweck C. S. (2005). Personal goals, dysphoria, & coping strategies. Unpublished manuscript, Columbia University.

[jir12970-bib-0004] Baird G. L. , Scott W. D. , Dearing E. & Hamill S. K. (2009) Cognitive self‐regulation in youth with and without learning disabilities: Academic self‐efficacy, theories of intelligence, learning vs. performance goal preferences, and effort attributions. Journal of Social and Clinical Psychology 28, 881–908.

[jir12970-bib-0005] Benjamini Y. & Hochberg Y. (2005) Controlling the false discovery rate: A practical and powerful approach to multiple testing. Journal of the Royal Statistical Society 57, 289–300.

[jir12970-bib-0006] Binning K. R. , Wang M.‐T. & Amemiya J. (2018) Persistence mindset among adolescents: Who benefits from the message that academic struggles are normal and temporary? Journal of Youth and Adolescence 48, 269–286. 30276598 10.1007/s10964-018-0933-3

[jir12970-bib-0007] Blackwell L. S. , Trzesniewski K. H. & Dweck C. S. (2007) Implicit theories of intelligence predict achievement across an adolescent transition: A longitudinal study and an intervention. Child Development 78, 246–263. 17328703 10.1111/j.1467-8624.2007.00995.x

[jir12970-bib-0008] Boden M. T. & Moos R. (2009) Dually diagnosed patients' responses to substance use disorder treatment. Journal of Substance Abuse Treatment 37, 335–345. 19540699 10.1016/j.jsat.2009.03.012 PMC3292216

[jir12970-bib-0009] Broda M. , Yun J. , Schneider B. , Yeager D. S. , Walton G. M. & Diemer M. (2018) Reducing inequality in academic success for incoming college students: A randomized trial of growth mindset and belonging interventions. Journal of Research on Educational Effectiveness 11, 317–338. 10.1080/19345747.2018.1429037 PMC10798796 38250254

[jir12970-bib-0101] Burgoyne A. P. , Hambrick D. Z. , Moser J. S. & Burt S. A. (2018) Analysis of a mindset intervention. Journal of Research in Personality 77, 21–30.

[jir12970-bib-0102] Burnette J. L. , O Boyle E. H. , VanEpps E. M. , Pollack J. M. & Finkel, E. J. (2013) Mind‐sets matter: A meta‐analytic review of implicit theories and self‐regulation. Psychological Bulletin 139, 655–701. 22866678 10.1037/a0029531

[jir12970-bib-0010] Calvete E. , Fernández‐Gonzalez L. , Orue I. , Echezarraga A. , Royuela‐Colomer E. , Cortazar N. et al. (2019) The effect of an intervention teaching adolescents that people can change on depressive symptoms, cognitive schemas, and hypothalamic‐pituitary‐adrenal axis hormones. Journal of Abnormal Child Psychology 47, 1533–1546. 30903540 10.1007/s10802-019-00538-1 PMC6650351

[jir12970-bib-0011] Campbell M. , Robertson A. & Jahoda A. (2014) Psychological therapies for people with intellectual disabilities: Comments on a matrix of evidence for interventions in challenging behaviour. Journal of Intellectual Disability Research 58, 172–188. 23106865 10.1111/j.1365-2788.2012.01646.x

[jir12970-bib-0103] De Ruiter K. P. , Dekker M. C. , Verhulst F. C. & Koot H. M. (2007) Developmental course of psychopathology in youths with and without intellectual disabilities. Journal of Child Psychology and Psychiatry 48, 498–507. 17501731 10.1111/j.1469-7610.2006.01712.x

[jir12970-bib-0014] De Wit, M. , Moonen, X. & Douma, J. (2011). Richtlijn effectieve interventies LVB: Aanbevelingen voor het ontwikkelen, aanpassen en uitvoeren van gedragsveranderende interventies voor jeugdigen met een licht verstandelijke beperking [Guideline effective interventions MBID: Recommendations for the development, adaptation and implementation of behavior based interventions for youth with mild to borderline intellectual disabilities]. Utrecht: Landelijk Kenniscentrum LVB.

[jir12970-bib-0015] Dearing R. L. , Barrick C. , Dermen K. H. & Walitzer K. S. (2005) Indicators of client engagement: Influences on alcohol treatment satisfaction and outcomes. Psychology of Addictive Behaviors 19, 71–78. 15783280 10.1037/0893-164X.19.1.71

[jir12970-bib-0104] Douma J. , Moonen X. , Noordhof L. & Ponsioen A. (2012). Richtlijn diagnostisch onderzoek LVB. Landelijk Kenniscentrum LVB.

[jir12970-bib-0017] Duckworth A. , Peterson C. , Matthews M. & Kelly D. (2007) Grit: Perseverance and passion for long‐term goals. Journal of Personality and Social Psychology 92, 1087–1101. 17547490 10.1037/0022-3514.92.6.1087

[jir12970-bib-0018] Dweck C. S. (1999) Self‐theories: Their role in motivation, personality, and development. Psychology Press, New York, NY.

[jir12970-bib-0105] Dweck C. S. & Leggett E. L. (1988) A social‐cognitive approach to motivation and personality. Psychological Review 95, 256–273.

[jir12970-bib-0019] Dweck C. S. & Yeager D. S. (2019) Mindsets: A view from two eras. Perspectives on psychological science: a journal of the Association for Psychological Science 14, 481–496. 30707853 10.1177/1745691618804166 PMC6594552

[jir12970-bib-0106] Einfeld S. L. , Ellis L. A. & Emerson E. (2011) Comorbidity of intellectual disability and mental disorder in children and adolescents: A systematic review. Journal of Intellectual & Developmental Disability 36, 137–143. 21609299 10.1080/13668250.2011.572548

[jir12970-bib-0022] Foliano, F. , Rolfe, H. , Buzzeo, J. , Runge, J. & Wilkinson, D. (2019). Changing mindsets: Effectiveness trial. National Institute of Economic and Social Research.

[jir12970-bib-0023] Helmond, P. , Verberg, F. & Overbeek, G. (2022). A pilot randomized controlled trial of the online mindset intervention The Growth Factory for youth with ID and/or mental health problems. [Manuscript submitted for publication]. Department of Child Development and Education, University of Amsterdam.

[jir12970-bib-0024] Heyvaert M. , Maes B. & Onghena P. (2010) A meta‐analysis of intervention effects on challenging behaviour among persons with intellectual disabilities. Journal of Intellectual Disability Research 54, 634–649. 20492347 10.1111/j.1365-2788.2010.01291.x

[jir12970-bib-0025] Hoyt C. L. , Forsyth R. B. & Burnette J. L. (2018) Social dominance orientation moderates the effectiveness of mindset messages. British Journal of Social Psychology 57, 448c460. 29359330 10.1111/bjso.12238

[jir12970-bib-0107] Kelly M. M. , Tyrka A. R. , Price L. H. & Carpenter L. L. (2008) Sex differences in the use of coping strategies: Predictors of anxiety and depressive symptoms. Depression and Anxiety 25, 839–846. 17603810 10.1002/da.20341 PMC4469465

[jir12970-bib-0026] Koestner R. , Aube J. , Ruttner J. & Breed S. (1995) Theories of ability and the pursuit of challenge among adolescents with mild mental retardation. Journal of Intellectual Disability Research 39, 57–65. 7719063 10.1111/j.1365-2788.1995.tb00914.x

[jir12970-bib-0027] Limeri L. B. , Carter N. T. , Choe J. , Harper H. G. , Martin H. R. , Benton A. et al. (2020) Growing a growth mindset: Characterizing how and why undergraduate students' mindsets change. International Journal of STEM Education 7, 35.

[jir12970-bib-0028] MacKinnon D. P. , Fairchild A. J. & Fritz M. S. (2007) Mediation analysis. Annual Review of Psychology 58, 593–614. 10.1146/annurev.psych.58.110405.085542 PMC2819368 16968208

[jir12970-bib-0108] Miller S. D. , Bargmann S. , Chow D. , Seidel J. & Maeschalck C. (2016) Feedback‐informed treatment (FIT): Improving the outcome of psychotherapy one person at a time. In: Quality improvement in behavioral health (eds W. O'Donohue & A. Maragakis ), pp. 247–262. Springer International Publishing.

[jir12970-bib-0029] Miu A. S. & Yeager D. S. (2015) Preventing symptoms of depression by teaching adolescents that people can change: Effects of a brief incremental theory of personality intervention at 9‐month follow‐up. Clinical Psychological Science 3, 726–743.

[jir12970-bib-0031] Mrazek A. J. , Ihm E. D. , Molden D. C. , Mrazek M. D. , Zedelius C. M. & Schooler J. W. (2018) Expanding minds: Growth mindsets of self‐regulation and the influences on effort and perseverance. Journal of Experimental Social Psychology 79, 164–180.

[jir12970-bib-0032] Muthén L. K. & Muthén B. O. (1998‐2012) Mplus User's Guide, 7th edn. Muthén & Muthén, Los Angeles, CA.

[jir12970-bib-0033] Paunesku D. , Walton G. M. , Romero C. , Smith E. N. , Yeager D. S. & Dweck C. S. (2015) Mind‐set interventions are a scalable treatment for academic underachievement. Psychological Science 26, 784–793. 25862544 10.1177/0956797615571017

[jir12970-bib-0109] Schleider J. L. & Weisz J. R. (2018) A single‐session growth mindset intervention for adolescent anxiety and depression: 9‐month outcomes of a randomized trial. Journal of Child Psychology and Psychiatry 59, 160–170. 28921523 10.1111/jcpp.12811

[jir12970-bib-0110] Schleider J. L. , Abel M. R. & Weisz J. R. (2015) Implicit theories and youth mental health problems: A random‐effects meta‐analysis. Clinical Psychology Review 35, 1–9. 25462109 10.1016/j.cpr.2014.11.001

[jir12970-bib-0034] Schleider J. L. , Burnette J. L. , Widman L. , Hoyt C. & Prinstein M. J. (2020) Randomized trial of a single‐session growth mind‐set intervention for rural adolescents' internalizing and externalizing problems. Journal of Clinical Child and Adolescent Psychology 49, 660–672. 31219698 10.1080/15374416.2019.1622123 PMC6923626

[jir12970-bib-0036] Schleider J. L. & Weisz J. R. (2016a) Mental health and implicit theories of thoughts, feelings, and behavior in early adolescents: Are girls at greater risk? Journal of Social and Clinical Psychology 35, 130–151.

[jir12970-bib-0037] Schleider J. L. & Weisz J. R. (2016b) Reducing risk for anxiety and depression in adolescents: Effects of a single‐session intervention teaching that personality can change. Behaviour Research and Therapy 87, 170–181. 27697671 10.1016/j.brat.2016.09.011 PMC5127737

[jir12970-bib-0038] Schulz K. F. , Altman D. G. & Moher D. (2010) CONSORT 2010 statement: Updated guidelines for reporting parallel group randomised trials. International Journal of Surgery 9, 672–677. 10.1016/j.ijsu.2011.09.004 22019563

[jir12970-bib-0111] Shirk S. R. & Saiz C. C. (1992) Clinical, empirical, and developmental perspectives on the therapeutic relationship in child psychotherapy. Developmental Psychopathy 4, 713–728.

[jir12970-bib-0039] Sisk V. F. , Burgoyne A. P. , Sun J. , Butler J. L. & Macnamara B. N. (2018) To what extent and under which circumstances are growth mind‐sets important to academic achievement? Two meta‐analyses. Psychological Science 29, 549–571. 29505339 10.1177/0956797617739704

[jir12970-bib-0040] Verberg F. , Helmond P. , Otten R. & Overbeek G. (2019) Mindset and perseverance of adolescents with intellectual disabilities: Associations with empowerment, mental health problems, and self‐esteem. Research in Developmental Disabilities 91, Article 103426. 31252179 10.1016/j.ridd.2019.103426

[jir12970-bib-0041] Verberg F. , Helmond P. , Otten R. & Overbeek G. (2021) Effectiveness of the online mindset intervention ‘The Growth Factory’ for adolescents with intellectual disabilities. Journal of Applied Research in Intellectual Disabilities 35, 217–230. 34608719 10.1111/jar.12941

[jir12970-bib-0042] Verberg F. , Helmond P. & Overbeek G. (2018) Study protocol: A randomized controlled trial testing the effectiveness of an online mindset intervention in adolescents with intellectual disabilities. BMC Psychiatry 18, 377. 30514245 10.1186/s12888-018-1939-9 PMC6278007

[jir12970-bib-0043] Verhulst F. C. & Van der Ende J. (2013). Handleiding ASEBA. Vragenlijsten voor leeftijden 6 tot en met 18 jaar. [ASEBA Manual. Questionnaires for ages 6 to 18 years]. Rotterdam: ASEBA Nederland.

[jir12970-bib-0112] Yeager D. S. & Dweck C. S. (2012) Mindsets that promote resilience: When students believe that personal characteristics can be developed. Educational Psychologist 47, 302–314.

[jir12970-bib-0044] Yeager D. S. & Dweck C. S. (2020) What can be learned from growth mindset controversies? American Psychologist 75, 1269–1284. 33382294 10.1037/amp0000794 PMC8299535

[jir12970-bib-0045] Yeager D. S. , Johnson R. , Spitzer B. J. , Trzesniewski K. H. , Powers J. & Dweck C. S. (2014) The far‐reaching effects of believing people can change: Implicit theories of personality shape stress, health, and achievement during adolescence. Journal of Personality and Social Psychology 106, 867–884. 24841093 10.1037/a0036335

[jir12970-bib-0046] Yeager D. S. , Romero C. , Paunesku D. , Hulleman C. S. , Schneider B. , Hinojosa C. et al. (2016) Using design thinking to improve psychological interventions: The case of the growth mindset during the transition to high school. Journal of Educational Psychology 108, 374–391. 27524832 10.1037/edu0000098 PMC4981081

[jir12970-bib-0047] Yeager D. S. , Trzesniewski K. H. & Dweck C. S. (2013) An implicit theories of personality intervention reduces adolescent aggression in response to victimization and exclusion. Child Development 84, 970–988. 23106262 10.1111/cdev.12003 PMC3660787

[jir12970-bib-0048] Yeager D. S. , Trzesniewski K. H. , Tirri K. , Nokelainen P. & Dweck C. S. (2011) Adolescents' implicit theories predict desire for vengeance after peer conflicts: Correlational and experimental evidence. Developmental Psychology 47, 1090–1107. 21604865 10.1037/a0023769

[jir12970-bib-0049] Yeager D. S. & Walton G. M. (2011) Social‐psychological interventions in education: They're not magic. Review of Educational Research 81, 267–301.

[jir12970-bib-0050] Zainal N. H. & Newman M. G. (2019) Relation between cognitive and behavioral strategies and future change in common mental health problems across 18 years. Journal of Abnormal Psychology 128, 295–304. 31045412 10.1037/abn0000428 PMC6707366

[jir12970-bib-0051] Zhang Z. , Gerstein D. R. & Friedmann P. D. (2008) Patient satisfaction and sustained outcomes of drug abuse treatment. Journal of Health Psychology 13, 388–400. 18420772 10.1177/1359105307088142 PMC2796692

[jir12970-bib-0052] Zhou Q. , Hofer C. , Eisenberg N. , Reiser M. , Spinrad T. L. & Fabes R. A. (2007) The developmental trajectories of attention focusing, attentional and behavioral persistence, and externalizing problems during school‐age years. Developmental Psychology 43, 369–385. 17352545 10.1037/0012-1649.43.2.369 PMC1832154

