# Supplementary material for: The online mindset intervention ‘The Growth Factory’ for adolescents with intellectual disabilities: moderators and mediators
Source: J Intellect Disabil Res. 2022 Sep 1;66(10):817–32. doi: 10.1111/jir.12970 (PMC9543548; doi:10.1111/jir.12970)
Supplement: Supplementary file 1 — Data S1. Supporting Information [file JIR-66-817-s001.doc]

**CONSORT-SPI 2018 Checklist**

|  | **SECTION** |  |  | **ITEM #** |  |  | **CONSORT-SPI 2010** |  |  | **CONSORT-SPI** |  |  | **REPORTED ON PAGE** |  |
| --- | --- | --- | --- | --- | --- | --- | --- | --- | --- | --- | --- | --- | --- | --- |
|  |  |  |  |  |  |  |  |  |  |  |  |  |  |  |
|  |  |  |  |  |  |  |  |  |  | **2018** |  |  | **#** |  |
|  |  |  |  |  |  |  |  |  |  |  |  |  |  |  |
|  | **TITLE AND ABSTRACT** | |  |  |  |  |  |  |  |  |  |  |  |  |
|  |  |  |  | 1a | |  | Identification as a randomised trial in | |  |  |  |  |  | |
|  |  |  |  |  |  |  | the title§ | |  |  |  |  |  | |
|  |  |  |  |  |  |  |  |  |  |  |  |  |  |  |
|  |  |  |  |  |  |  | Structured summary of trial design, | |  | Refer to CONSORT extension for | |  | 14 | |
|  |  |  |  | 1b | |  | methods, results, and conclusions (for | |  | social and psychological | |  |  |  |
|  |  |  |  |  |  |  | specific guidance see CONSORT for | |  | intervention trial abstracts | |  |  | |
|  |  |  |  |  |  |  |  |  |  |  |  |  |  |  |
|  |  |  |  |  |  |  | Abstracts)§ | |  |  |  |  |  |  |
|  | **INTRODUCTION** | |  |  |  |  |  |  |  |  |  |  |  |  |
|  |  |  |  | 2a | |  | Scientific background and explanation | |  |  |  |  | 5-8 | |
|  |  |  |  |  |  |  | of rationale § | |  |  |  |  |  | |
|  | Background and | |  |  |  |  |  |  |  |  |  |  |  |  |
|  |  |  |  |  |  |  |  |  |  | If pre-specified, how the | |  |  |  |
|  | Objectives | |  |  |  |  |  |  |  |  |  |  | 8 | |
|  |  |  |  | 2b | |  | Specific objectives or hypotheses § | |  | intervention was hypothesied to | |  |  |  |
|  |  |  |  |  |  |  |  |  |  |  |  |  |  |  |
|  |  |  |  |  |  |  |  |  |  | work | |  |  |  |
|  |  |  |  |  |  |  |  |  |  |  |  |  |  |  |
|  | **METHODS** | |  |  |  |  |  |  |  |  |  |  |  |  |
|  |  |  |  |  |  |  | Describe of trial design (such as | |  | If the unit of random assignment is | |  | 9 | |
|  |  |  |  |  |  |  |  |  |  | not the individual, please refer to | |  |  |  |
|  |  |  |  | 3a | |  | parallel, factorial), including allocation | |  |  |  |  |  |  |
|  |  |  |  |  |  |  |  |  |  | CONSORT for Cluster Randomized | |  |  | |
|  |  |  |  |  |  |  | ratio § | |  |  |  |  |  |  |
|  | Trial Design | |  |  |  |  |  |  |  | Trials | |  |  |  |
|  |  |  |  |  |  |  |  |  |  |  |  |  |  |  |
|  |  |  |  |  |  |  | Important changes to methods after | |  |  |  |  | 10 | |
|  |  |  |  | 3b | |  | trial commencement (such as eligibility | |  |  |  |  |  |  |
|  |  |  |  |  |  |  |  |  |  |  |  |  |  |  |
|  |  |  |  |  |  |  | criteria), with reasons | |  |  |  |  |  |  |
|  |  |  |  |  |  |  |  |  |  |  |  |  |  |  |
|  |  |  |  |  |  |  |  |  |  | When applicable, eligibility criteria | |  | 10 | |
|  |  |  |  | 4a | |  | Eligibility criteria for participants§ | |  | for settings and those delivering the | |  |  |  |
|  |  |  |  |  |  |  |  |  |  |  |  |  |  |  |
|  | Participants | |  |  |  |  |  |  |  | interventions | |  |  |  |
|  |  |  |  |  |  |  |  |  |  |  |  |  |  |  |
|  |  |  |  | 4b | |  | Settings and locations where the data | |  |  |  |  | 9 | |
|  |  |  |  |  |  |  | were collected | |  |  |  |  |  | |
|  |  |  |  |  |  |  |  |  |  |  |  |  |  |  |
|  |  |  |  |  |  |  | The interventions for each group with | |  |  |  |  | 9-12 | |
|  |  |  | 5 | |  |  | sufficient details to allow replication, | |  |  |  |  |  |  |
|  |  |  |  |  |  |  | including how and when they are | |  |  |  |  |  | |
|  |  |  |  |  |  |  |  |  |  |  |  |  |  |  |
|  | Interventions | |  |  |  |  | actually administered § | |  |  |  |  |  |  |
|  |  |  |  |  |  |  |  |  |  | Extent to which interventions were | |  |  | |
|  |  |  |  | 5a | |  |  |  |  | actually delivered by providers and | |  |  |  |
|  |  |  |  |  |  |  |  |  |  |  |  |  | 10, Figure 1 | |
|  |  |  |  |  |  |  |  |  |  | taken up by participants as planned | |  |  |  |
|  |  |  |  |  |  |  |  |  |  |  |  |  |  |  |

|  |  |  |  | Where other informational materials | 13 |
| --- | --- | --- | --- | --- | --- |
|  |  | 5b |  | about delivering the intervention |  |
|  |  |  |  |  |  |
|  |  |  |  | can be accessed |  |
|  |  |  |  |  |  |
|  |  |  |  | When applicable, how intervention | 9 |
|  |  | 5c |  | providers were assigned to each |  |
|  |  |  |  |  |  |
|  |  |  |  | group |  |
|  |  |  |  |  |  |
|  |  |  | Completely defined pre-specified |  | 11-14 |
|  |  | 6a | outcomes, including how and when |  |  |
|  |  |  |  |  |  |
|  | Outcomes |  | they were assessed§ |  |  |
|  |  |  |  |  |  |
|  |  | 6b | Any changes to trial outcomes after the |  |  |
|  |  |  | trial commenced, with reasons |  |  |
|  |  |  |  |  |  |
|  |  | 7a | How sample size was determined§ |  | 9 |
|  |  |  |  |  |  |
|  | Sample Size |  |  |  |  |
|  |  |  | When applicable, explanation of any |  |  |
|  |  | 7b | interim analyses and stopping |  |  |
|  |  |  |  |  |  |
|  |  |  | guidelines |  |  |
|  |  |  |  |  |  |
|  | **RANDOMISATION** |  |  |  |  |
|  |  | 8a | Method used to generate the random |  | 9 |
|  |  |  | allocation sequence |  |  |
|  | Sequence |  |  |  |  |
|  |  |  | Type of randomisation; detail of any |  |  |
|  | generation |  |  |  | 9 |
|  |  | 8b | restriction (such as blocking and block |  |  |
|  |  |  |  |  |  |
|  |  |  | size)§ |  |  |
|  |  |  |  |  |  |
|  |  |  | Mechanism used to implement the |  |  |
|  | Allocation concealment |  | random allocation sequence, describing |  | 9 |
|  |  | 9 | any steps taken to conceal the |  |  |
|  | mechanism |  |  |  |  |
|  |  |  | sequence until interventions were |  |  |
|  |  |  |  |  |  |
|  |  |  | assigned§ |  |  |
|  |  |  | Who generated the random allocation |  |  |
|  | Implementation | 10 | sequence, who enrolled participants, |  | 9, 10 |
|  |  |  | and who assigned participants to |  |  |
|  |  |  |  |  |  |
|  |  |  | interventions^§^ |  |  |
|  |  |  | Who was aware of intervention |  |  |
|  | Awareness of |  | assignment after allocation (for |  | 10 |
|  |  | 11a | example, participants, providers, those |  |  |
|  | assignment |  |  |  |  |
|  |  |  | assessing outcomes), and how any |  |  |
|  |  |  |  |  |  |
|  |  |  | masking was done |  |  |

|  |  | 11b | If relevant, description of the similarity |  |  |
| --- | --- | --- | --- | --- | --- |
|  |  |  | of interventions |  |  |
|  |  |  |  |  |  |
|  |  |  | Statistical methods used to compare | How missing data were handled, | 13, 14 |
|  |  | 12a |  | with details of any imputation |  |
|  |  |  | group outcomes§ |  |  |
|  | Analytical |  |  | method |  |
|  |  |  |  |  |  |
|  | methods |  | Methods for additional analyses, such |  |  |
|  |  | 12b | as subgroup analyses, adjusted |  |  |
|  |  |  |  |  |  |
|  |  |  | analyses, and process evaluations |  |  |
|  |  |  |  |  |  |
|  | **RESULTS** |  |  |  |  |
|  |  |  | For each group, the numbers randomly | Where possible, the number | Figure 1 |
|  |  | 13a | assigned, receiving the intended | approached, screened, and eligible |  |
|  | Participant flow (a |  | intervention, and analysed for the | prior to random assignment, with |  |
|  |  |  |  |  |  |
|  | diagram is strongly |  | outcomes§ | reasons for non-enrolment |  |
|  | recommended) |  | For each group, losses and exclusions |  | Figure 1 |
|  |  | 13b | after randomisation, together with |  |  |
|  |  |  |  |  |  |
|  |  |  | reasons§ |  |  |
|  |  |  |  |  |  |
|  |  | 14a | Dates defining the periods of |  | 9, Figure 1 |
|  |  |  | recruitment and follow-up |  |  |
|  | Recruitment |  |  |  |  |
|  |  | 14b | Why the trial ended or was stopped |  |  |
|  |  |  |  |  |  |
|  |  |  |  |  |  |
|  |  |  |  |  |  |
|  | Baseline data | 15 | A table showing baseline | Include socioeconomic variables | Tables 1 and 2 |
|  |  |  | characteristics for each group§ | where applicable |  |
|  |  |  |  |  |  |
|  |  |  | For each group, number included in |  | Figure 1, Table 2 |
|  | Numbers analysed | 16 | each analysis and whether the analysis |  |  |
|  |  |  |  |  |  |
|  |  |  | was by original assigned groups§ |  |  |
|  |  |  |  |  |  |
|  |  |  | For each outcome, results for each | Indicate availability of trial data |  |
|  |  | 17a | group, and the estimated effect size |  | 15, 16, Figure 1-4, Tables 1-4 |
|  |  |  | and its precision (such as 95% |  |  |
|  | Outcomes and |  |  |  |  |
|  |  |  | confidence interval)§ |  |  |
|  | estimation |  |  |  |  |
|  |  |  | For binary outcomes, the presentation |  |  |
|  |  |  |  |  |  |
|  |  | 17b | of both absolute and relative effect |  |  |
|  |  |  | sizes is recommended |  |  |
|  |  |  | Results of any other analyses |  |  |
|  |  |  | performed, including subgroup |  |  |
|  | Ancillary analyses | 18 | analyses, adjusted analyses, and |  |  |
|  |  |  | process evaluations, distinguishing pre- |  |  |
|  |  |  | specified from exploratory |  |  |

|  |  |  |  | All important harms or unintended |  |  |
| --- | --- | --- | --- | --- | --- | --- |
|  | Harms |  | 19 | effects in each group (for specific |  |  |
|  |  |  |  | guidance see CONSORT for Harms) |  |  |
|  | **DISCUSSION** | |  |  |  |  |
|  |  |  |  | Summarize the main results (including | Trial limitations, addressing sources |  |
|  |  |  |  | an overview of concepts, themes, and | of potential bias, imprecision, and, if | 17-20 |
|  | Limitations |  | 20 | types of evidence available), link to the | relevant, multiplicity of analyses |  |
|  |  |  |  |  |  |  |
|  |  |  |  | review questions and objectives, and |  |  |
|  |  |  |  |  |  |  |
|  |  |  |  | consider the relevance to key groups. |  |  |
|  | Generalisability |  | 21 | Discuss the limitations of the scoping | Generalisability (external validity, |  |
|  |  |  |  | review process. | applicability) of the trial findings§ |  |
|  |  |  |  |  |  |  |
|  |  |  |  | Provide a general interpretation of the | Interpretation consistent with | 17-20 |
|  | Interpretation |  | 22 | results with respect to the review | results, balancing benefits and |  |
|  |  |  |  | questions and objectives, as well as | harms, and considering other |  |
|  |  |  |  |  |  |  |
|  |  |  |  | potential implications and/or next steps. | relevant evidence |  |
|  | **IMPORTANT INFORMATION** | |  |  |  |  |
|  | Registration |  | 23 | Registration number and name of trial |  | 9, 22 |
|  |  |  |  | registry |  |  |
|  |  |  |  |  |  |  |
|  | Protocol |  | 24 | Where the full trial protocol can be |  | 23 |
|  |  |  |  | accessed, if available |  |  |
|  |  |  |  |  |  |  |
|  | Declaration of Interests |  | 25 | Sources of funding and other support; | Declaration of any other potential | 23 |
|  |  |  |  | role of funders | interests |  |
|  |  |  |  |  |  |  |
|  |  |  |  |  | Any involvement of the intervention | 23 |
|  |  |  | 26a |  | developer in the design, conduct, |  |
|  |  |  |  |  |  |  |
|  |  |  |  |  | analysis, or reporting of the trial |  |
|  | Stakeholder |  |  |  |  |  |
|  |  |  | 26b |  | Other stakeholder involvement in |  |
|  | investments |  |  |  |  |  |
|  |  |  |  |  | trial design, conduct, or analyses |  |
|  |  |  |  |  |  |  |
|  |  |  | 26c |  | Incentives offered as part of the trial |  |
|  |  |  |  |  |  |  |
|  |  |  |  |  |  |  |

This table lists items from the CONSORT 2010 checklist (with some modifications for social and psychological intervention trials) and additional items in the CONSORT-SPI 2018 extension. Empty rows in the ‘CONSORT-SPI 2018’ column indicate that there is no extension to the CONSORT 2010 item

*We strongly recommended that the CONSORT-SPI 2018 Explanation and Elaboration (E&E) document be reviewed when using the CONSORT-SPI 2018 checklist for important clarifications on each item

§An extension item for cluster trials exists for this CONSORT 2010 item

**Citations**

Montgomery, P., Grant, S., Mayo-Wilson, E., Macdonald, G., Michie, S., Hopewell, S., & Moher, D. (2018). Reporting randomised trials of social and psychological interventions: the CONSORT-SPI 2018 Extension. *Trials*, *19*(1), 407.

Grant, S., Mayo-Wilson, E., Montgomery, P., Macdonald, G., Michie, S., Hopewell, S., & Moher, D. (2018). CONSORT-SPI 2018 Explanation and Elaboration: guidance for reporting social and psychological intervention trials. *Trials*, *19*(1), 406.
